# General practitioners’ experiences of emergency care and treatment planning in England: a focus group study

**DOI:** 10.1186/s12875-021-01486-w

**Published:** 2021-06-24

**Authors:** Caroline J. Huxley, Karin Eli, Claire A. Hawkes, Gavin D. Perkins, Rob George, Frances Griffiths, Anne-Marie Slowther

**Affiliations:** 1 Warwick Medical School, University of Warwick, Gibbet Hill, Coventry, CV4 7AL UK; 2 St Christopher’s Hospice, 51-59 Lawrie Park Road, London, SE26 6DZ UK

**Keywords:** Primary health care, Emergency care and treatment plans, DNACPR, End of life care, Recommended Summary Plan for Emergency Care and Treatment, ReSPECT

## Abstract

**Background:**

Emergency Care and Treatment Plans are recommended for all primary care patients in the United Kingdom who are expected to experience deterioration of their health. The Recommended Summary Plan for Emergency Care and Treatment (ReSPECT) was developed to integrate resuscitation decisions with discussions about wider goals of care. It summarises treatment recommendations discussed and agreed between patients and their clinicians for a future emergency situation and was designed to meet the needs of different care settings. Our aim is to explore GPs’ experiences of using ReSPECT and how it transfers across the primary care and secondary care interface.

**Methods:**

We conducted five focus groups with GPs in areas being served by hospitals in England that have implemented ReSPECT. Participants were asked about their experience of ReSPECT, how they initiate ReSPECT-type conversations, and their experiences of ReSPECT-type recommendations being communicated across primary and secondary care. Focus groups were transcribed and analysed using Thematic Analysis.

**Results:**

GPs conceptualise ReSPECT as an end of life planning document, which is best completed in primary care. As an end of life care document, completing ReSPECT is an emotional process and conversations are shaped by what a ‘good death’ is thought to be. ReSPECT recommendations are not always communicated or transferable across care settings. A focus on the patient’s preferences around death, and GPs’ lack of specialist knowledge, could be a barrier to completion of ReSPECT that is transferable to acute settings.

**Conclusion:**

Conceptualising ReSPECT as an end of life care document suggests a difference in how general practitioners understand ReSPECT from its designers. This impacts on the transferability of ReSPECT recommendations to the hospital setting.

**Supplementary Information:**

The online version contains supplementary material available at 10.1186/s12875-021-01486-w.

## Introduction

The Recommended Summary Plan for Emergency Care and Treatment (ReSPECT) is an Emergency Care and Treatment Plan (ECTP), developed to integrate Do Not Attempt Cardiopulmonary Resuscitation (DNACPR) decisions with discussions about wider goals of care [1, 2]. ECTPs were designed to address concerns identified about the use of standalone DNACPR decisions by contextualising them within broader treatment plans making recommendations for use in future emergency situations. Key issues with standalone DNACPR orders included a conflation of the term ‘DNACPR’ with limitations on other forms of treatment [3–5], geographical inconsistencies in recording decisions including recognition of different forms across healthcare organisations [6, 7], and evidence that people with DNACPR decisions receive poorer care than those without [8–10]. In community settings many patients were dying without a recorded DNACPR decision [11]. Reasons for this included GPs waiting until the patient has deteriorated before addressing DNACPR; having anxieties about discussing resuscitation with patients and their families; and attempting to avoid conflict [12].

ReSPECT was designed to travel with the patient and be recognised across different care settings. It records treatment recommendations discussed and agreed between the patients and their clinicians for a future emergency situation when patients may not have capacity to make decisions for themselves. This study is part of a wider mixed-method NIHR-funded study evaluating how ReSPECT is implemented in acute care [13]. The present study explores the use of ReSPECT in primary care in areas served by hospitals using ReSPECT. Our aim is to explore experiences of the ReSPECT process in the community, and how it transfers across the primary and secondary care interface.

## Method

### Design and setting

We conducted focus groups with general practitioners in England, in areas served by four acute hospital trusts that implemented ReSPECT and participated in the wider ReSPECT Evaluation Study. The hospitals were self-selecting early adopters and the involvement of general practice in ReSPECT implementation varied across the sites, from a fully integrated implementation plan to simply informing local practices of the new policy.

Our approach was experiential and we worked within a critical realist paradigm underpinned by the assumptions that participants’ language reflects reality, but this reality is mediated by the interpretations of our participants and ourselves [14]. This study was reviewed and approved by the Coventry and Warwickshire Research Ethics Committee (reference number 17/WM/0134).

### Participant recruitment and characteristics

The study team engaged in snowball sampling. We sent information about the research to key contacts in areas served by four hospital trusts participating in the wider study. These key contacts included Principal Investigators at the hospital sites, Primary Care Clinical Research Networks, a GP partnership, a GP interest group, a health and care commissioning group, and a palliative care specialist with GP contacts. These key contacts facilitated recruitment in their networks by sharing letters of invitation and the participant information sheets, and placing adverts in newsletters. The study was originally designed to include only GPs; however, based on initial findings and local PI feedback, we expanded the recruitment criteria to include other healthcare professionals. Due to limitations related to the timescale for amending the study, the additional healthcare professionals could not be included in the focus groups and were interviewed separately, 4 months after the focus group data collection ended. This presented both methodological and temporal differences (in-person focus groups versus online interviews), and as a result, we have decided to limit the analysis in this paper to the focus groups. Participants were paid £150 for their time and were offered CPD certificates for participation. Five focus groups of between three and ten participants were conducted. Two focus groups were served by the same acute hospital Trust.

### Data collection

Written informed consent was obtained from participants before commencing each focus group discussion. All focus groups were facilitated by KE, a medical anthropologist, and four were co-facilitated by CAH, a health services researcher. In two focus groups, the facilitators were joined by a palliative care specialist from the local hospital, who responded to participants’ locally-specific questions about ReSPECT. At the start of each group facilitators circulated a ReSPECT form and described the ReSPECT process. Although working in areas served by hospitals using ReSPECT, some GPs had not had any experiences of this process prior to the focus group. Therefore, GPs’ discussion about ReSPECT was informed by their experience of other forms (DNACPR and specialised local forms).

A topic guide developed for this study (see Supplementary Files) explored participants’ experiences of ReSPECT (both completing it themselves, and seeing their patients discharged from hospital with ReSPECT), how they initiated ReSPECT-type conversations, and their experiences of ReSPECT-type recommendations being communicated across the primary care-secondary care interface. Focus groups were conducted between April and November 2019 and lasted between 60 and 105 min. They were audio recorded, and recordings were transcribed verbatim.

### Data analysis

Transcripts were analysed using inductive thematic analysis [15]. CJH, a psychologist, read the transcripts in an immersion process, closely coded them at the semantic-level and identified numerous candidate themes. These were discussed with KE and CAH to ensure candidate themes reflected issues observed within the focus groups, as well as themes KE noted in a preliminary analysis of the first three focus group transcripts, conducted to inform a planned study of ReSPECT in the community. Discussion of candidate themes within the research team identified key issues to focus on; how GPs conceptualised ReSPECT (an issue which was inductively identified within the data), and how ReSPECT-type recommendations translated across settings (an issue which was derived from the aim of the study). Transcripts were re-coded to check for data on these topics. Following this second round of coding five themes were identified. These were refined through further team discussion resulting in four themes and one subtheme. In our analysis, editing of data extracts is indicated with a bracketed gap, and all identifying features were removed to ensure anonymity.

## Results

Twenty-seven participants took part in the focus groups, all of whom were GPs. Participants in three focus groups had used ReSPECT in their own clinical practice, while participants in the other two groups had not (see Table 1).

**Table 1:** Focus groups participants’ experience of ReSPECT

Focus Group (*n*)	Is ReSPECT used in participants’ GP practices?	Number of months ReSPECT had been used by their local hospital at the time of the focus group
FG1 (*n* = 10)	Yes	27
FG2 (*n* = 3)	No	23
FG3 (*n* = 5)	No	23
FG4 (*n* = 4)	Yes	27
FG5 (*n* = 5)	Yes	27

### ReSPECT is an end of life care document

There was an implicit assumption that ReSPECT was to be used to plan end of life care, and should be used for patients who were “*coming to the last 2 or 3 years of their life”* (FG5); palliative care patients, frail patients, or patients in the final stages of a chronic illness*.* Participants found it easier to initiate RESPECT conversations with advanced cancer patients (with clear illness trajectories) than patients who were frail or were living with a chronic health condition (such as COPD or heart failure) where the prognosis was less certain and the patient themselves had little awareness of their potential trajectory.

ReSPECT-type conversations were predominantly initiated by the GPs themselves, but sometimes by other health professionals, such as Macmillan nurses. The trigger for such conversations was typically a deterioration in the patient’s health*.* For palliative patients, this might mean a change in Gold Standard Framework (GSF) classification, from green to amber, or amber to red*.* For patients with dementia the trigger for a ReSPECT conversation would be the diagnosis itself. Participants felt it was important to identify and record the patient’s wishes while they had capacity*.* ReSPECT was also initiated as part of a routine care home admission.

Several participants had a “*hunch*” or they “*just knew*” that it was the right time to initiate a ReSPECT conversation*.* Their feeling was sometimes prompted by verbal cues (such as references to recent experiences in hospital) that could be used as an opening to the conversation. An existing relationship with the patient enabled them to know when the time was right.

Less common was the patient themselves initiating a ReSPECT-type conversation*.* The focus tended to be resuscitation, with the patients certain they did not want to be resuscitated in the event of an emergency. Patients who wanted to formalise a desire to decline CPR were often healthy, and our participants questioned the morality of doing a ReSPECT-type form for people who were not obviously approaching end of life: “*some patients will want a DNACPR in place when there’s really nothing wrong with them […] they just don’t like the idea of going through resuscitation. Then, that’s a whole other minefield […] am I really doing the right thing for this person when they could have a really positive outcome potentially with treatment?”* (FG2).

#### ReSPECT is best done in primary care

There was a general consensus that ReSPECT-type conversations could be done well within primary care. Conversations were often planned for home visits with the patient aware that they will discuss end of life care, thus ensuring that the patient was as prepared and comfortable as possible. Some GPs had lengthy pre-existing relationships with their patients, and this made the conversation easier for both the GP and the patient: *“you do get to know them, you do get to know the family and build that relationship. So it does make sort of discussions like this much easier, ‘cause you’ve built that relationship with them”* (FG1).

ReSPECT-type discussions were described as an ongoing process which takes time to complete; they were “*not a one off conversation”* (FG3). This process involved judging when the time was right to start the discussion, holding initial conversations, and ensuring family members were present when decisions were made*.* As patients were not usually at immediate risk of deterioration, the availability of time was seen as real “*advantage*” the GPs had over acute care. Because of these perceived advantages afforded by primary care participants felt that they should be the ones holding ReSPECT conversations, rather than hospital staff*.*

GPs described time and resource constraints as barriers to ReSPECT-type conversations. If they identified a patient approaching end of life within their usual clinic, the GPs felt constrained by the 10 min consultation slot and aware of a busy waiting room outside: “*I think when you’re reaching them, they already come to you for something else. And they’re in there within 5 or 10 min, you can’t really”* (FG1). Our participants also pointed out that while they knew many patients, they did not know everyone. They were reluctant to hold sensitive and emotional ReSPECT-type conversations with someone they had not met before because of the lack of rapport and knowledge about the patient. A lack of experience and confidence in end of life planning also prevented GPs from initiating ReSPECT-type conversations.

While participants reported barriers to completing ReSPECT in primary care, they ultimately thought that it was the place to hold such conversations, and were critical of some of the ReSPECT forms they had seen completed in hospitals. The GPs were sympathetic that the busy hospital environment was not well suited to in-depth conversations about a patient’s end of life care. They also thought that forms were being completed by junior doctors who were inexperienced, and who were “*not necessarily savvy to what language is best at home”* (FG5) where the use of medical jargon could confuse and worry patients.

### ReSPECT is an emotional process

Many GPs reported gauging how emotionally prepared a patient was to have the conversation by assessing their reaction when the topic was raised. If the patient reacted with alarm or withdrawal, the GP delayed the conversation until a later date. They described these initial contacts as “*warning shots*” to give the patient time to emotionally prepare themselves.

Many GPs planned a ReSPECT-type conversation for a time when the patient’s family could be included, to provide emotional support and ensure that everyone understood the plan regarding the patient’s end of life. Although inclusion of the patient’s family created potential for conflict, participants suggested that the relationship between themselves and the family could be improved by holding such conversations: “*generally once you’ve had that discussion and you’ve reached a decision, it can actually be quite, quite a positive relationship going forwards. And families are very, very grateful for that input […] They’re incredibly grateful for the time that you put in with them”* (FG2).

A small number of participants reported not feeling emotionally affected themselves by ReSPECT-type conversations. This was rationalised as being a consequence of experience. Additionally, the GPs typically had these conversations with older patients approaching end of life, and while they acknowledged this was sad they felt less psychological burden than when having such conversations with younger patients.

More frequently, the GPs found it hard to maintain an emotional distance, particularly when they knew the patient well. The GP’s emotional reaction could be affected by the patient’s reaction to the conversation. If the patient reacted positively GPs felt they had “*done a good thing*” (FG3), and felt a sense of pride in the process. However, distressed patients left the GPs feeling upset, having gone on the “*journey*” with them.

### Conversations are driven by cultural understandings of death

Participants held implicit understandings of what a “*good death*” was for their patients. Typically this was “*peacefully*” with no CPR or invasive treatment, either at home or in a hospice. This death was described as something that patients usually wanted for themselves; their “*best case scenario*”. As GPs were holding ReSPECT-type conversations with patients they expected to die fairly soon, these understandings often underpinned their conversations and the medical recommendations they recorded: “*we've discussed […] whether they don’t want to be admitted to hospital, just die peacefully at home or die, want to die in the hospice”* (FG4). Some GPs were aware that their understandings were culturally-bound. They described how in some cultures a ‘good death’ meant trying to maintain life for as long as possible, rather than focussing on quality of life: “*the main thing that culturally they would want to, to kind of, keep that person going and, and give them the best chance possible […] as opposed to thinking, “Hang on, about, what, let’s think about the quality of their life left,” and I think that’s a big cultural, thing”* (FG3).

GPs’ understandings of a ‘good death’ for their patients sometimes conflicted with the wishes of the patient themselves. Typically, disagreements were around resuscitation when patients (or their family) wanted CPR attempted and the GPs felt this was inappropriate. During such conflict our participants would try to nudge the patient/family in the specific direction that they thought appropriate. For example, participants described how patients did not understand what resuscitation meant, and they would stress that there was no guarantee that CPR would work*.* Others described in blunt terms what resuscitation would entail, persuading patients that it was inappropriate: *“What do you want [paramedics] to do? Do you want them to push your husband away and assault you? Or do you want them to check that your heart has stopped? And then put an arm around your husband and make him a cup of tea?”* (FG5). Participants observed that patients often believed that if they agreed not to be resuscitated, they would receive no treatment or care at all. They described reassuring patients that they would still receive good care but it sometimes took several consultations for the GPs to explain why resuscitation or hospital admission would not be in the patient’s best interest.

GPs noted that families often struggled to discuss end of life care. They felt “*scared*” of using terms such as ‘death’ or ‘dying’ and felt that by raising the topic they were “*condemning*” their relative. Participants suggested that they should be ones to initiate the conversations and use phrases such as “*when you die*” to remove that burden from families*.* Our participants theorised that resistance to discussing death was grounded in fear. It was acknowledged too that doctors can be fearful and subtly feed into patients’ fear by reinforcing the need to take medication to prevent death: “*I think sometimes as doctors, there can be a fear about death. And sometimes we’re the biggest culprit for that, and kind of feeding into patients’ ideas that you can’t die, and you’ve just kind of gotta keep taking the tablets, keep alive”* (FG2). A few participants were keen to break down taboos surrounding discussion of death in order to normalise decisions about end of life care. They suggested that holding ReSPECT-type discussions earlier, on first diagnosis or as part of a routine check-up, would help to normalise it for both themselves and for their patients.

### There can be difficulties translating ReSPECT across care settings

GPs gave examples where ReSPECT recommendations had translated into care. For example, paramedics had used ReSPECT to decide whether to transport a patient to hospital or not. However, they also described situations when the recommendations recorded on ReSPECT were not transferred into care. Generally this involved patients being admitted to hospital, despite a recorded preference for non-admission. This was sometimes because of a lack of service availability, such as home care support services or hospice beds*.* Sometimes, patients were transferred into hospital because their family could no longer cope with caring for them at home. Several participants mentioned that translating ReSPECT recommendations into care could be difficult in nursing homes because occasionally the staff-to-client ratio meant that if a client deteriorated then healthcare staff would be limited in the emotional and physical support they could provide: “*you’re the only nurse looking after 80 clients overnight and one goes off it’s actually really difficult. Even if it says not for resuscitation, not for admission, you’re the only nurse, what does that actually mean, how do you kind of support?”* (FG1).

The GPs were aware that their lack of knowledge of specialist interventions and treatments available within acute care could be a barrier to completing a ReSPECT form that was meaningful in hospital. One participant suggested that ReSPECT should be initiated in secondary care and reinforced in primary care, because GPs “*would struggle to have that detailed conversation”* (FG3). The GPs talked about focussing in more general terms on preferences around hospital admission and resuscitation, and on treating chronic or terminal illness rather than emergencies. The ReSPECT form was seen as an important document that patients should take with them to hospital, whatever the cause of admission. Participants were keen that, having gone through a lengthy process with the patient, ReSPECT recommendations should be used to inform care. However, they acknowledged that it is difficult for any health professional not to actively treat patients: “*you’ve got somebody who’s palliative care, advanced cancer, bed bound, falls out breaks their leg, and goes in and, and absolutely everything gets scanned from head to toe […] they may well have expressed that that’s not what they wanted. It’s very difficult not to just treat the bit that needs treating”* (FG1).

Some GPs had not seen hospital-issued ReSPECT forms even though their patients’ discharge letters mentioned ReSPECT. These participants expected to see a copy of the ReSPECT form with the discharge letters and felt disadvantaged by this apparent lack. They were unclear whether the form was kept with the patient or in hospital records. Participants felt that an electronic version transferable between settings would be beneficial “*so that all the different people providing care for a particular patient have got the […] same kind of document that they can resort to in terms of palliative care and patient’s wishes” *(FG4). To gain a digital copy of the form they had completed, GPs created workarounds, such as manually transferring ReSPECT recommendations to their electronic records in the surgery. These tended to increase their workload and diverged from the intended usage (hard copy held by patients).

When our participants had seen hospital-issued ReSPECT forms, they thought that they were too focussed on specific treatments available in hospital, or were used as replacement DNACPR forms. Important but uncomfortable topics, such as where a person wanted to die, were not discussed: “*They might deal with […] IV, antibiotics, fluids. But they don’t properly discuss, like, hospice or, you know, things, where you want your end of life to be, and, which are a bit more challenging, I think, for us to discuss” (FG4).* These comments suggest that ReSPECT could be being used for different purposes in primary and acute settings.

## Discussion

GPs conceptualised ReSPECT as an end of life care document, to be used as part of advance care planning. ReSPECT was originally designed as an emergency care plan providing concise recommendations for treatment for a future emergency situation [1, 16]. While the two are complementary they have historically been separate [1]; advance care plans are detailed and often focus specifically on end of life care, whereas emergency plans are more concise and provide recommendations for use in any kind of medical emergency [16]. Conceptualising ReSPECT as an end of life document suggests a difference in how general practitioners understand ReSPECT from its designers, and suggests there may be differences in how ReSPECT is being used in primary and acute settings. For GPs the focus is end of life care and whether the patient wants to be admitted to hospital or not. For hospital doctors the focus is treatment in hospital if the patient deteriorates while in their care [17]. These conflicting aims mean that the treatments and care described on respective ReSPECT forms might not be useful in different settings.

Our research indicates some similarities between GPs and hospital doctors in how they experience ReSPECT. Like hospital doctors [17], our participants initiated conversations when the time was ‘right’ and when they had time to have the conversation. Time pressures are experienced by doctors in both settings, however GPs have an advantage in being able to plan home visits for ReSPECT-type conversations, where they have protected time with few distractions. Both groups described a process of multiple conversations before arriving at an agreed decision [17]. However, there was a difference in terms of urgency; within hospitals, ReSPECT conversations are conducted with people who are expected to deteriorate within a short time frame when the patient is still under hospital care [17], in contrast there is less immediate urgency in the community. Moreover, whereas hospital doctors initiated ReSPECT with patients with the highest risk of deteriorating further during the admission, GPs initiated ReSPECT conversations in response to deterioration that had already happened.

Our participants’ descriptions of a ‘good death’ reflected current Western ideals; peaceful, dignified, and free from pain, however, these ideals might not represent all patients’ understandings of a ‘good death’ [18]. Patients are sometimes pressured into making decisions that correspond with these Western ideals [18], and our participants described trying to nudge the patient/family in a specific direction, typically around CPR, potentially breaching patient autonomy.

Our findings suggest that more training should be provided in primary care on the wider use of ReSPECT, to expand its usage beyond end of life care. GPs should be aware that ReSPECT-type conversations take time in order to be done well. As GPs experience time constraints as a barrier, these conversations could more frequently involve other healthcare professionals who have existing relationships with the patient and their family. For example, Macmillan and district nurses work closely with patients and already hold informal conversations which could be formalised through ReSPECT. Communication of ReSPECT forms between primary and secondary care could also be improved, potentially through electronic communication of ReSPECT recommendations, with a timeframe for GP review (i.e. is GP review urgently needed or not).

Research is needed to fully evaluate the use of ReSPECT in the community, including the experiences of other community-based healthcare professionals and the impact of ReSPECT on outcomes for primary care patients. More research is also needed on how ReSPECT transfers across the primary-secondary care interface, particularly examining how useful ReSPECT recommendations made in one setting are in another setting. Research should also explore the impact of culture on the suitability of ReSPECT for people from different backgrounds. Finally, as ReSPECT was usually only initiated for people approaching the final year of their life, exploration of when patients think is the best time to hold such discussions would be beneficial to inform effective implementation in primary care. A forthcoming study evaluating the use of ReSPECT in primary care has recently been funded by NIHR HS&DR programme commenced in May 2021 [19].

### Limitations

This is the first study providing insight into how ReSPECT is being used in primary care. We recruited our participants from diverse geographical areas, including urban centres and rural areas, however it is limited to primary care in England.

Our study is limited by our participant sample. Not all participants were using ReSPECT in their practice, so their understandings of the process or how it was intended to be used might not have been fully formed. However our sample provides a diversity of experience of ReSPECT which reflects the reality of its introduction by different organizations and their approaches to coordinating its introduction between primary and secondary care. Our participants may have self-selected for the research because of an interest in palliative care, which would contribute to the conceptualisation of ReSPECT as an end of life document. We did not record participants’ backgrounds, however in one focus group several participants mentioned they were the palliative care lead for their practice or had an interest in palliative care. The limitations of our participant sample could be a result of the snowball sampling method used for recruitment. Finally, only GPs were included in the focus groups, which limits the transferability of the findings.

While candidate themes were discussed within the research team to ensure that they reflected the dataset, we did not have capacity to engage in secondary coding to cross-check findings. Finally, due to the coronavirus pandemic, we were unable to conduct focus groups in one hospital area, included in the other arms of our research, where ReSPECT had been implemented later.

## Conclusion

ReSPECT is conceptualised by GPs as an end of life planning document, which is best completed in primary care. ReSPECT was designed to be recognised within different care settings, however, ReSPECT-type recommendations, for many reasons, are not always communicated or transferable across primary and acute services. It is possible that ReSPECT is being conceptualised and used for different purposes in different settings.

## Supplementary Information

**Additional file 1.** ReSPECT Evaluation: GP Focus Group Topic Guide.

## Data Availability

The datasets generated and/or analysed during the current study are not publicly available due to the possibility that participant privacy may be compromised, but are available from the corresponding author on reasonable request.

## Abbreviations

ReSPECT: Recommended Summary Plan for Emergency Care and Treatment
ECTP: Emergency Care and Treatment Plan
DNACPR: Do Not Attempt Cardiopulmonary Resuscitation
