# Supplementary material for: General practitioners’ experiences of emergency care and treatment planning in England: a focus group study
Source: BMC Fam Pract. 2021 Jun 24;22:128. doi: 10.1186/s12875-021-01486-w (PMC8224258; doi:10.1186/s12875-021-01486-w)
Supplement: Supplementary file 1 — Additional file 1. ReSPECT Evaluation: GP Focus Group Topic Guide. [file 12875_2021_1486_MOESM1_ESM.docx]

**ReSPECT Evaluation: GP Focus Group Topic Guide**

1. Have you had any experiences with the ReSPECT form?
   1. Were you ever involved in completing a ReSPECT form?
   2. Have any of your patients been discharged from hospital or returned from an outpatient hospital appointment with a ReSPECT form?
   3. Were you involved with the ReSPECT process in other ways?
   4. Has there been any discussion of ReSPECT in your commissioning group?
2. Is the ReSPECT form used at your practice? If so, when has ReSPECT been implemented at your practice?
   1. How frequently do you complete ReSPECT forms as part of your practice?
   2. How are patients made aware of ReSPECT at your surgery (e.g. via notice boards, leaflets in the waiting room)?
   3. Has any of your patients initiated a conversation with you about ReSPECT?
   4. Are any other community-based practitioners with whom you work involved in ReSPECT discussions?
   5. Has your practice or patient involvement group provided feedback on the implementation of ReSPECT?
3. If you do not use the ReSPECT form at your practice, do you use other forms to conduct conversations about emergency care and recommend particular courses of action?
4. When do you usually initiate a ReSPECT (or advanced care planning) conversation?
   1. What circumstances make it more likely for you to initiate this conversation with particular patients?
   2. What circumstances make it less likely?
   3. Do you gauge patients’ emotional readiness for the ReSPECT conversation before starting the discussion? If so, how?
5. In your perspective, who should be involved in the ReSPECT conversation?
   1. What is the role of the patient’s family in the ReSPECT conversation?
6. How do you start a typical ReSPECT (or advanced care planning) conversation?
   1. Where do you situate the CPR discussion within the ReSPECT conversation? How does that affect the rest of the conversation?
7. In many ReSPECT conversations, patients are asked to imagine a difficult future scenario. How do you present these future scenarios to your patients and their relatives?
   1. Do you sometimes doubt the ability of patients and relatives to imagine these potential scenarios? In these cases, what do you do?
   2. Should a patient who prefers not to know what their future holds be asked to imagine these scenarios?
8. During the ReSPECT conversation, what approaches do you use to maintain rapport with patients? With patients’ families?
   1. Does the ReSPECT form influence your discussions with patients and families?
   2. Do you think the ReSPECT conversation affects the doctor-patient relationship? If so, in what ways?
   3. How do you make sure that patients and families understand what has been discussed and decided in the ReSPECT conversation?
   4. How do you make patients aware that they can change or update their decisions?
9. How do you experience the communication of ReSPECT decisions across the primary care / acute care divide? Are there gaps in communication, and if so, where (and why)?
   1. What strategies do you use to overcome these communication gaps?
10. How do you perceive the impact of the ReSPECT process on patient and family experiences, and on the relationships between patients and GPs?
    1. In your experience, how do ReSPECT forms issued in hospital (as opposed to primary care) impact on patients, their relatives, and their relationship with GPs and other community health care providers?
11. What effect does completing a ReSPECT form have on you?
    1. What challenges have you encountered in conducting ReSPECT conversations and completing ReSPECT forms?
    2. Has the ReSPECT form affected how you structure your decision-making process? If so, how?
12. How can the ReSPECT process and/or form can be improved to meet the needs of primary care settings?
